# A Rac1-FMNL2 signaling module affects cell-cell contact formation independent of Cdc42 and membrane protrusions

**DOI:** 10.1371/journal.pone.0194716

**Published:** 2018-03-26

**Authors:** Hanna Grobe, Andrea Wüstenhagen, Christian Baarlink, Robert Grosse, Katharina Grikscheit

**Affiliations:** Institute of Pharmacology, Biochemical-Pharmacological Center, Philipps-University of Marburg, Marburg, Germany; University of Illinois at Chicago, UNITED STATES

## Abstract

*De novo* formation of epithelial cell-cell contacts relies on actin-based protrusions as well as tightly controlled turnover of junctional actin once cells encounter each other and adhesion complexes assemble. The specific contributions of individual actin regulators on either protrusion formation or junctional actin turnover remain largely unexplored. Based on our previous findings of Formin-like 2 (FMNL2)-mediated control of junctional actin dynamics, we investigated its potential role in membrane protrusions and impact on newly forming epithelial contacts. CRISPR/Cas9-mediated loss of FMNL2 in human MCF10A cells combined with optogenetic control of Rac1 activity confirmed its critical function in the establishment of intercellular contacts. While lamellipodial protrusion rates remained unaffected, FMNL2 knockout cells were characterized by impaired filopodia formation similar to depletion of the Rho GTPase Cdc42. Silencing of Cdc42, however, failed to affect FMNL2-mediated contact formation. Hence, we propose a cell-cell contact-specific and Rac1-mediated function of FMNL2 entirely independent of Cdc42. Consistent with this, direct visualizations of native epithelial junction formation revealed a striking and specifically Rac1- and not Cdc42-dependent recruitment of FMNL2 to newly forming junctions as well as established cell-cell contacts within epithelial sheets.

## Introduction

Protrusive membrane structures such as filopodia or lamellipodia are important mediators of cellular motility and are critically involved in cell migration, tumor cell invasion or epithelial differentiation [1, 2]. The formation of cellular protrusions relies on highly organized and tightly controlled rearrangements of the actin cytoskeleton in space and time. By controlling and guiding the activity of a diverse group of actin nucleators and assembly factors, the family of small Rho GTPases takes center stage in directing the remodeling of the actin cytoskeleton [3]. This particularly involves the activities of formin proteins and the Arp2/3 complex, which are differentially orchestrated by the two GTPases Rac1 and Cdc42 to promote outgrowth of cellular protrusions, with Rac1 being dominantly involved in the formation of lamellipodia and Cdc42 to mainly regulate filopodia growth [4].

While the contribution of actin-mediated protrusions downstream of Rho GTPases has been extensively studied during cell migration, the functions of these exploratory structures during the formation of epithelial cell-cell contacts remains less well understood. Previous studies using MDCK cells revealed the extension of Rac1-dependent lamellipodia in cells facing each other, which upon encounter initiate novel cell-cell contact sites characterized by subsequent lateral expansion and accumulation of the adhesion receptor E-Cadherin [5, 6]. Noteworthy, this reorganization of cell-cell adhesions was shown to coincide with a substantial rearrangement of the actin cytoskeleton at newly forming junctions [6]. Other studies highlighted the importance of filopodia in the establishment of cell-cell contacts showing that primary mouse keratinocytes extend filopodial structures enriched for E-cadherin at their tips to contact neighboring cells. These filopodia generate so-called “adhesion zippers” which eventually develop further into mature intercellular adhesions [7]. Consistently, both lamellipodia and filopodia could be observed at the leading edge during dorsal closure in Drosophila [8] allowing to speculate on a potential interplay of these distinct cellular protrusions during the process of epithelialization.

Besides *de novo* cell-cell contact formation also maturation and maintenance of intercellular adhesions are directly affected by the spatial organization and turnover of junctional actin to efficiently adapt to constantly changing requirements in epithelial homeostasis [9–11]. However, the exact mechanisms controlling actin dynamics during the different steps of epithelial junction formation remain to be fully understood.

Members of the formin family represent the largest group of Rho GTPase effectors being capable of potent remodeling of the actin cytoskeleton through activities involving actin filament nucleation, elongation or filament bundling to various extents [12–14]. Several formins have been linked to the formation as well as maintenance of cell-cell junctions in various cell and animal models [15]. Of note, two members of the formin-like (FMNL) subgroup, namely FMNL2 and FMNL3, have been detected at filopodial as well as lamellipodial protrusions and were shown to affect cell-cell contact formation through their impact on junctional actin [16–21]. Using human breast epithelial MCF10A cells, we recently identified a critical role of FMNL2 in the assembly of junctional actin at newly forming cell-cell contacts in a 3D matrix [21]. This activity originates downstream of Rac1 and is in line with a physical association of FMNL2 and components of the cadherin-catenin complex [21]. Other studies provided evidence for an interaction between FMNL2 and active Cdc42 and link FMNL2 functions to lamellipodial protrusions during migration of B16-F1 melanoma cells [16, 22, 23]. Whether the physiological functions of FMNL2 during contact formation can be attributed to protrusive membrane structures or involve GTPases other than Rac1 warrants further investigation.

Here, we dissect functions and upstream regulations of FMNL2 at epithelial cell-cell contact sites. We employ optogenetics to directly visualize epithelial junction formation in real-time and generated FMNL2 knockout cells allowing to test for specific defects during the initiation of intercellular adhesions. Loss of FMNL2 resulted in delayed and overall impaired formation of cell-cell contacts consistent with a distinct recruitment and enrichment of FMNL2 to contact sites and an association of FMNL2 to the C-terminal region of E-Cadherin. Both, FMNL2 function and localization at the cell-cell interface involve activity of Rac1, but occur independent of Cdc42. Whereas we found FMNL2 and Cdc42 to affect filopodia formation, we did not observe a significantly impaired extension of lamellipodia in cells lacking FMNL2. Together, this suggests that neither alterations in filopodia nor lamellipodia dynamics are likely to account for the impaired establishment of cell-cell contacts. In contrast, our findings reinforce the presence of a cell-cell adhesion-specific signaling module involving the formin FMNL2 to establish epithelial architecture downstream of Rac1.

## Material and methods

### Reagents, antibodies, and plasmids

Cell culture reagents were purchased from Invitrogen. Other reagents were from Sigma-Aldrich if not stated otherwise. Restriction enzymes and PCR reagents were purchased from Thermo Scientific. Antibodies used were from Sigma-Aldrich (rabbit anti-FMNL2 HPA005464); Cell Signaling Tech (rabbit anti-tubulin 11H10, rabbit anti-Cdc42 #2462, rabbit anti-Rac1 #24655, rabbit anti N-WASP #4848); BD Biosciences (mouse anti–E-Cadherin #610182). Goat anti-WAVE1 was from R&D System (kindly provided by Kuner lab). Rabbit anti-FMNL1 and rabbit anti-FMNL3 were kindly provided by D. Billadeau (Mayo Clinic, Rochester). Secondary antibodies were purchased from Bio-Rad Laboratories, Inc. (anti-rabbit-HRP), GE Healthcare (anti–mouse-HRP) or JacksonImmuno (anti-goat).

All FMNL2 derivatives used refer to human FMNL2. Full-length FMNL2 (aa 1–1,092), FMNL2 CT (aa 521–1,092), or FMNL2 NT (aa 23–484) were cloned into pEF-FLAG or pEF-myc vectors (previously described in [24]). FMNL2-NT-GFP comprises FMNL2 amino acids 1–484 and cloned into pEGFP-N1 (Clontech) and was further subcloned into pWPXL (primers used: fw 5’ gcgcgcgtttaaactaatgggcaacgcagggagcatgg 3’, rev 5’ gcgcgcactagtctacttgtacagctcgtccatgccgagagt 3’). Fascin cDNA was amplified from MCF10A cells and cloned to a pEF backbone containing an N-terminal GFP tag (pEF-GFP using ClaI / SpeI (fw 5’ atatatatcgataccgccaacggcacagccgag 3’ and rev 5’ atatatactagtctagtactcccagagcgaggcggg). GFP-Fascin was further subcloned into pWPXL using using MssI/SpeI (5’ gcgcgcgtttaaactatggtgagcaagggcgaggagc 3’). The pEGFP-C2-α-catenin plasmid (gift from E. Sahai, Cancer Research UK) was subcloned into pWPXL using MssI/SpeI restriction sites (fw 5’ gcgcgcgtttaaactaatggtgagcaagggcgaggag 3’, rev 5’ gcgcgcactagttcagatgctgctgtccatggctttgaac 3’). pInducer20-BFP-Cdc42 N17 is based on GFP-Cdc42 N17 (M. Way, Cancer Research UK) and was generated by an overlapping PCR using the following primer (Overlap primer 5’ aaactggggcacaagcttaatggacagacaattaagtgtgttgttgtg 3’, fw BFP 5’ gcgcgcggatccggatgagcgagctgattaagga 3’, rev Cdc42 5’ gcgcgcctcgagttagaatatacagcacttcc 3’). BFP-Cdc42 N17 was cloned into pENTR11 via BamHI/XhoI restriction sites. pENTR11-BFP-Cdc42 was used to allow recombination into pINDUCER20 according to the manufacturer’s protocol (Thermo Fisher). The pInducer20 plasmid was kindly provided by G. Hu (National Institute of Environmental Health Sciences, Research Triangle Park, NC) [25]. Lentiviral pINDUCER20-E-Cadherin-GFP, pWPXL-FMNL2-GFP and pWXPL-mCherry-PA-Rac1-L61 were described in [21, 26].

### Cell culture, 3D cell culture, WST assay, SRF luciferase assay

Human breast epithelial MCF10A cells (ATCC) were cultured at 37°C and 5% CO_2_ atmosphere in MCF10A growth medium (DMEM/F12 supplemented with 5% horse serum, 20 ng/ml EGF, 0.5% hydrocortisone, 100 ng/ml cholera toxin, 10 ug/ml insulin, 1% penicillin-streptomycin) as described previously [21, 27]. HEK 293 and HEK 293T cells (ATCC) were maintained in DMEM supplemented with 10% FCS. For long-term 3D cell culture, MCF10A cells were seeded into 3-D Life Hydrogel supplemented with RGD peptides (Cellendes) into 8-well μ-slides (Ibidi) (3,000 cells per 8-well) [21]. Cells were cultured for 14 days with medium changes every second day (MCF10A assay medium containing 5 ng/ml EGF). After 14 days cells were fixed with 8% Formaldehyde for 10 minutes, washed with PBS and permeabilized with 0.3% Triton X-100/PBS for 10 minutes. After washing, cells were stained with DAPI (1:10,000 in PBS) and Phalloidin-488 (Thermo Fisher). Per condition, 20–30 spheroids were counted. WST proliferation assays were performed according to the manufacturer’s instructions (WST-1; Roche). Per well 5.000 cells were seeded in triplicates. SRF luciferase reporter gene assays were performed as described in [28, 29].

### Western Blotting and co-immunoprecipitation

Standard Western blotting and co-immunoprecipitation were performed as in [21]. In short, transfected HEK 293 cells were lysed in RIPA buffer (50 mM Tris, 150 mM NaCl, 2 mM EDTA, 0.1% Triton X-100, 0.25% DOC and 0.1% SDS) supplemented with protease inhibitors (Roche) and incubated with M2-FLAG beads (Sigma Aldrich) for 1 h at 4°C. Eluted proteins were subjected to Western blotting using either FLAG-HRP (Sigma Aldrich) or GFP-HRP (Thermo Fisher) antibodies.

### Transfection, lentiviral transduction, and siRNA knockdown

Transfections of HEK 293/T cells and lentiviral transduction of MCF10A cells were performed as in [21]. In brief, HEK 293T cells were transfected using the calcium phosphate method with packaging plasmids (pSPAX and pMDG.2) and the pWPXL or pINDUCER20 plasmids. After 48 hours, virus-containing supernatant was filtered and incubated with MCF10A cells overnight. To ensure homogenous expression of fluorescent proteins, cells were further sorted using FACS. siRNAs were purchased from QIAGEN and transfected using Lipofectamine RNAiMax (Thermo Scientific) according to the manufacturer’s protocol. The following Flexitube siRNAs were used: HS_FMNL2_6 (5′-CAAATTAGGCCTGGACGAATA3′), HS_FMNL2_7 (5′-TGGGACTAGATGGCCCACTAA-3′), HS_CDC42 (5’-CATCAGATTTTGAAAATATTTAA3′), HS_CDH1_12 (5′-CTAGGTATTGTCTACTCTGAA-3′), HS_RAC1_6 (5′-ATGCATTTCCTGGAGAATATA-3′) and negative control siRNA (5′-AATTCTCCGAACGTGTCACGT-3′). For photoactivation experiments, cells were seeded into 6-well plates (150.000 c/well) and transfected the next day. The following day, cells were seeded either onto glass-bottom dishes for live-cell imaging or for Western blotting and analyzed after 24 hours. For inhibition of Arp2/3 activity, subconfluent MCF10A monolayers were incubated with 10 μM CK-666 (Calbiochem, San Diego, CA) for 30 min prior to live-cell imaging.

### CRISPR/Cas9, sequencing, T7 assay

CRISPR (clustered regularly interspaced short palindromic repeats) /Cas9 genome editing was performed using the pSpCas9 (BB)-2A-GFP plasmid [30]. Short guide (sg) RNAs against FMNL2 exon 2 were designed using the crispr.mit.edu platform provided by the Zhang lab. SgRNA sequences were modified according to [30] (for FMNL2 exon 2: 5’ caccgatcatactgccgcagtaacc 3’ and 5’ aaacggttactgcggcagtatgatc 3’). SgRNAs were annealed using T4 PNK for 30 min at 37°C, 5 min at 95°C and cooled down to RT over 3 hours.

Diluted annealed sgRNA pairs were introduced to the pSpCas9 (BB)-2A-GFP plasmid through digestion/ligation cycles using BpII and T7 DNA ligase. SgRNAs were tested in HEK 293 cells for efficiency before transfection into MCF10A cells using Lipofectamine LTX (Thermo Fisher). After 24 hours, MCF10A cells were FACS-sorted for GFP expression and seeded into 96-well plates. Single clones were analyzed for protein knockdown and subsequently further characterized. For sequencing and T7 assays, we generated primers located in the intron sequence around exon 2 of FMNL2 (fw 5’ gcgcgcaagcttgtctgattgttcaaag 3’ and 5’ gcgcgcctcgagccacacaagtttagaggc 3’) to generate appropriately large fragments including restriction sites for cloning (HindIII/XhoI). After PCR and digestion, fragments were cloned into pCDNA.3 and five obtained clones were sent for sequencing. For T7 assays fragments were denatured, annealed and incubated with T7 endonuclease.

### Image acquisition, live-cell imaging, and photoactivation

All microscopic images were acquired using a confocal microscope (Zeiss LSM 700/800) equipped with a 63x/1.4 NA oil objective lens. All live-cell imaging experiments were carried out in an incubation chamber at 37°C and 5% CO_2_ atmosphere (Pecon). For live-cell imaging, MCF10A cells were seeded (100.000 cells) onto 35mm glass bottom dishes (MatTek Corporation) and imaged the following day. Optogenetic activation of PA-Rac1 was performed as previously described in [21]. In short, mCherry-PA-Rac1 was activated using the 488 nm laser, either on the overall area of imaging or within confined regions. Channels (GFP, mCherry or BFP) were recorded for maximal 150 frames at 5 s second intervals to ensure an apparent link to the stimulation of Rac1 and to avoid detrimental effects for the cells.

### Image analysis and statistics

Multiimage projection images of z-stacks or splitting of channels were done using the Zeiss Zen software. Images were adjusted using Adobe Photoshop. Quantifications and image analysis were performed using Fiji [31] or ImageJ [32] including the ‘kymographbuilder module’. Filopodia length was measured using ImageJ. Cell size changes and speed of contact formation were quantified using the open-source software ‘CellProfiler’ [33]. Statistical analyses were performed using Prism 6 (Graph Pad Prism Inc) or ‘R’ (R core team, 2016). Statistical significance was calculated using an unpaired students t-test to compare two groups or two-way ANOVA to compare three groups or more.

## Results

### CRISPR/Cas9-mediated knockout of FMNL2 confirms its impact on epithelial differentiation

**Fig 1:**
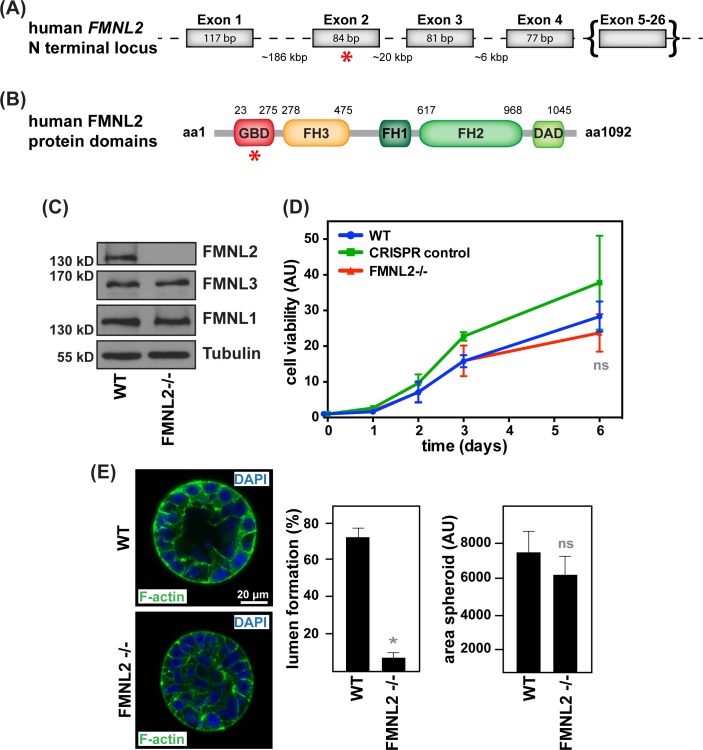
CRISPR/Cas9-mediated knockout of FMNL2 affects lumen formation in MCF10A cells. **(A)** Simplified structure of the human FMNL2 locus. **(B)** Cartoon illustrating the FMNL2 protein. Red asterisk (*) indicates the region targeted by Cas9. bp, base pairs; aa, amino acid; GBD, GTPase binding domain; FH, formin homology domain; DAD, diaphanous autoinhibitory domain. **(C)** Western Blot comparing FMNL1, 2, 3 expression in WT and FMNL2-/- cells. Antibodies were applied as indicated. **(D)** Colorimetric cell viability assay shows no differences in proliferation between generated cell lines (N = 3, triplicates for each time point, error bars SD, ns non-significant). **(E)** WT and FMNL2-/- cells were grown in hydrogel for 14 d before staining for F-actin and DAPI. Graphs depict quantifications of successful lumen formation and the overall area covered by spheroids (N = 3, *p ≤0.0001, *n* = 8, error bars SD, ns indicates no significance).

To generate MCF10A cells devoid of FMNL2, we targeted exon 2 of the corresponding human gene encoding for its N-terminal GTPase binding domain (GBD) using the CRISPR/Cas9 technology (Fig 1A and 1B, S1A Fig) [30]. Fig 1C confirms specific loss of endogenous FMNL2 expression in the obtained cell line without changes in the expression of the closely related formins FMNL1 and FMNL3. Sequencing of the targeted locus revealed a single nucleotide deletion in at least one allele of the FMNL2 gene resulting in a frame shift and premature stop codon at amino acid position 63 (S1B–S1D Fig). As a control, we simultaneously generated a monoclonal cell line undergoing the same procedure of genome editing, but lacking a guide RNA and therefore specific targeting of Cas9 endonuclease activity.

We performed a cell viability assay to exclude significant differences in overall growth or proliferation between wildtype, FMNL2-/- or CRISPR control cells (Fig 1D). When cultured in a 3D surrounding, control MCF10A cells form spheroids containing a characteristic lumen [27]. Although growing to similar sizes this lumen formation was severely impaired in spheroids lacking FMNL2 (Fig 1E) confirming our previous findings obtained by shRNA-mediated silencing of FMNL2 [21]. This reinforces a critical role of FMNL2 during epithelial differentiation.

### FMNL2 localizes to and affects newly forming epithelial cell-cell contacts

**Fig 2:**
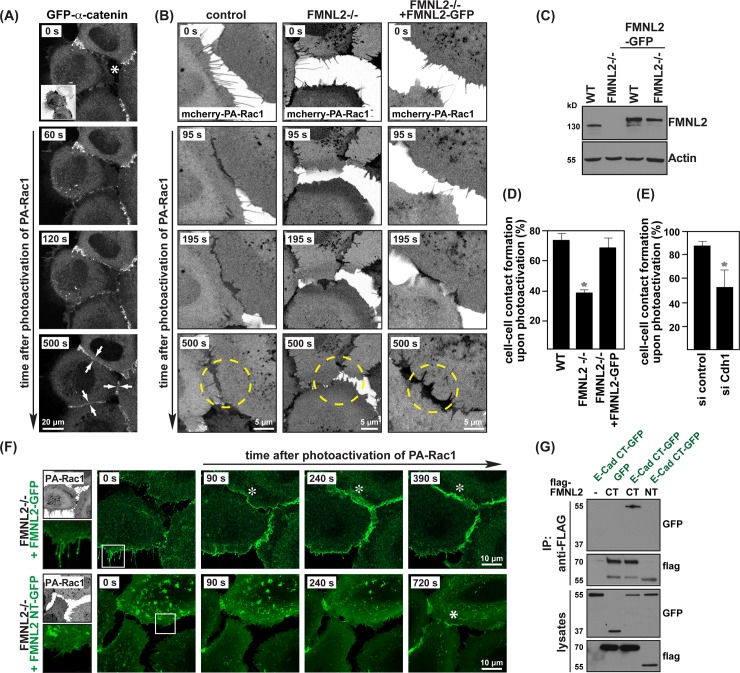
Impairment in cell-cell contact formation in FMNL2-/- cells can be rescued by reintroduction of FMNL2-GFP. **(A)** Live wildtype MCF10A cells expressing mCherry-PA-Rac1 and GFP-α-catenin were imaged over time to visualize adhesion junction formation upon Rac1 activation. Small inset shows mCherry-PA-Rac1 expression. **(B)** Comparison of cell-cell contact formation dynamics upon PA-Rac1 activation in indicated cell lines. Dashed circles highlight regions of interest. **(C)** Western blot comparing endogenous FMNL2 and exogenous FMNL2-GFP expression. Antibodies were applied as indicated. **(D)** Quantification of cell-cell contact establishment after Rac1 activation as in (B) (*n* = 60 (WT), *n* = 65 (FMNL2-/-), *n* = 64 (FMNL2-/- + FMNL2-GFP), pooled from three independent experiments, error bars SD, *p ≤0.05,). **(E)** Quantification of cell-cell contact formation in cells treated either with control or Cdh1 siRNA (*n* = 43 (si control), *n* = 44 (si Cdh1) pooled from three independent experiments, error bars SD, *p≤0.5 calculated by *t*-test). Western blot demonstrating siRNA efficiency in MCF10A cells in S1E Fig. **(F)** Live FMNL2-/- cells co-expressing FMNL2-GFP or FMNL2-NT-GFP with PA-Rac1 (upper small image) were imaged. Stills show protrusion dynamics and contact formation over time. Zoom image depict magnification of filopodia. Asterisks highlight areas of contact sites. **(G)** Co-immunoprecipitation of HEK cells expressing GFP or E-Cadherin CT-GFP with flag-tagged FMNL2 derivatives. Flag-tagged proteins were precipitated and bound proteins were detected using GFP-HRP antibody. Please note, E-Cadherin CT-GFP is pulled down only with flag-FMNL2 CT. In immunoprecipitates of cells expressing high levels of flag-FMNL2 CT, a band at 60 kD is observed likely deriving from a truncation product.

We previously made use of optogenetic control of Rac1 activity (PA-Rac1) to trigger intercellular contacts between adjacent cells [21, 26]. Here, we confirmed the successful formation of adhesive junctions by a specific recruitment of the junctional component α-catenin upon light-regulated activation of Rac1 (Fig 2A). This approach to stimulate and directly visualize *de novo* epithelial contact formation on demand allowed us to recapitulate the basic requirement of FMNL2 during this process.

FMNL2 knockout cells showed a significant impairment in establishing cell-cell contacts during the observed timeframe (Fig 2B–2D), comparable to cells silenced for the adhesion receptor E-Cadherin (Fig 2E). While Rac1-induced membrane protrusions rapidly form a nascent cell-cell contact in control cells, membrane protrusion in FMNL2-/- cells expand, but show delayed and overall impaired contact reinforcement. Importantly, this defect could be fully restored by the reintroduction of FMNL2-GFP into the FMNL2 -/- cells (Fig 2B–2D).

When we assessed the localization of FMNL2-GFP in MCF10A FMNL2-/- cells in the absence of cell-cell contacts, we observed enrichment at the plasma membrane as well as to the tips of filopodia (Fig 2F, upper panel), consistent with previous reports on different cell types [16, 20, 34]. Upon Rac1-induced contact formation, however, FMNL2-GFP underwent a rapid and striking redistribution towards newly forming cell-cell contacts where it remained throughout subsequent junction maturation (Fig 2F, S1 Movie). Membrane targeting of FMNL2 is suggested to be mediated by a combination of GTPase binding, N-terminal myristoylation as well as a basic patch residing in its N-terminal region [22, 35]. To test whether these basic requirements were sufficient for a specific subcellular targeting of FMNL2, we generated a GFP fusion of its isolated N-terminus (FMNL2 NT, aa 1–484) and stably expressed this variant in FMNL2 -/- cells (Fig 2F). Despite the presence of all aforementioned modules and although being able to reconstitute FMNL2 autoinhibition (S1F Fig), we could not detect an enrichment of FMNL2 NT to the tips of filopodia (Fig 2F). Expression of FMNL2 NT, lacking catalytic activity, failed to restore the impaired cell engagement of FMNL2 -/- cells which is consistent with a requirement of FMNL2-driven actin assembly during nascent contact formation (Fig 2F, S2 Movie).

We previously reported a Rac1-dependent physical interaction between endogenous FMNL2 and E-Cadherin [21]. Here, we extended this analysis and further confirmed that this association is mediated by the C-terminus of FMNL2 (aa 521–1092) and the C-terminus of E-Cadherin (aa 733–882) (Fig 2G). Therefore, failure of FMNL2 NT to restore a loss of endogenous FMNL2 might not only derive from its lack of catalytic activity, but also from an inability to interact with E-Cadherin. However, the exact role of the FMNL2/E-Cadherin interplay remains to be further elucidated. Of note, and in contrast to a lack of accumulation to filopodia tips, FMNL2 NT-GFP still showed enrichment at eventually developing cell-cell contacts (Fig 2F, latest time point) arguing for an alternative targeting mechanism involved and against a critical role of E-Cadherin binding in this process.

Taken together, FMNL2 NT therefore highlights a differential and most likely context-dependent control of FMNL2 subcellular targeting which appears to reach beyond its mere ability to associate to lipid membranes or to interact with certain Rho GTPases, potentially involving its dimerization, phosphorylation or its interaction to so far unknown partners [16, 21, 36].

#### Loss of FMNL2 does not affect lamellipodia outgrowth

**Fig 3:**
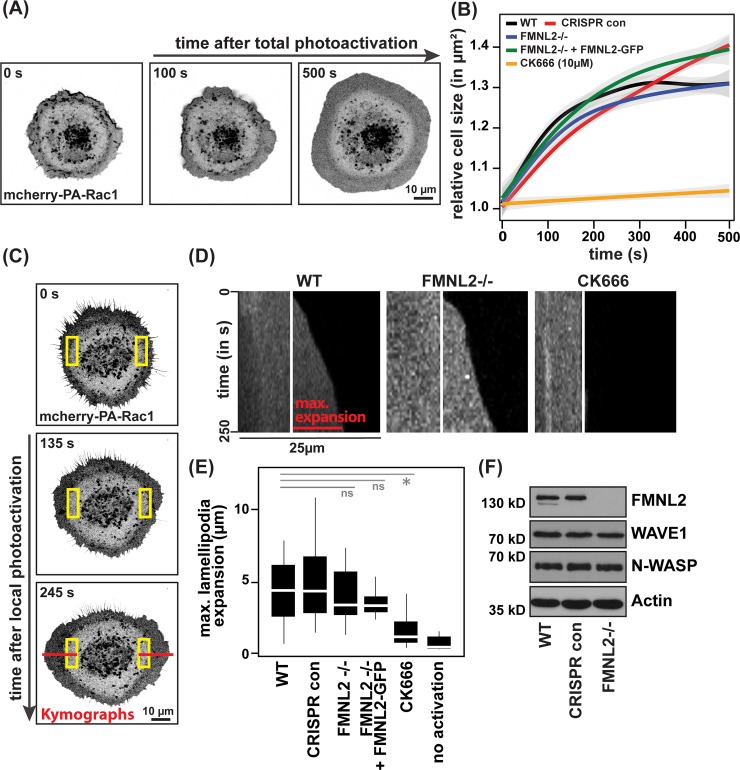
Rac1-induced lamellipodia formation is independent of FMNL2. **(A)** Single MCF10A cells expressing mCherry-PA-Rac1 were globally photoactivated and monitored over time. **(B)** Quantification of Rac1-induced cell enlargement in indicated cell lines over time (*n* = 15 (WT), *n* = 17 (CRISPR con), *n* = 15 (FMNL2-/-, *n* = 11 (FMNL2-/- + FMNL2-GFP), *n* = 3 (CK-666), data pooled from three independent experiments). Background displays 95% confidence interval of the regression. **(C)** MCF10A cells expressing mCherry-PA-Rac1 were photoactivated within indicated areas using the 488 nm laser (yellow boxes) and monitored over time. Kymographs were generated as indicated by red lines. **(D)** Examples of kymographs. **(E)** Quantification of maximum expansion of lamellipodia (*n* = 30 (WT), *n* = 24 (CRIPSR con), *n* = 18 (FMNL2-/-), *n* = 18 (FMNL2-/- + FMNL2-GFP), *n* = 16 (CK-666), *n* = 13 (not photoactivated), pooled from three independent experiments, *p≤ 0.001). **(F)** Western blot comparing expression levels of lamellipodial proteins in control and FMNL2-/- cells as indicated.

To address the phenotype of defective epithelial junction formation in the absence of FMNL2 in more detail, we next tested FMNL2-/- cells for a potentially altered ability to extend lamellipodial protrusions to reach their neighbors. For this, we first compared the overall extent and rate of circumferential lamellipodial protrusions, which we induced by the photoactivation of total cellular PA-Rac1 in single MCF10A cells (Fig 3A). Quantifications of the extent of cellular spreading over time revealed no significant differences between wildtype, CRISPR control or FMNL2 -/- cells (Fig 3B). In contrast, pretreatment with the Arp2/3 inhibitory compound CK-666, shown to affect lamelliopodia formation [37, 38], robustly prevented protrusive responses arising from light-induced activation of PA-Rac1 (Fig 3B). To better reflect the polar nature of a lamellipodium emerging at the front of migrating cells, we spatially restricted photoactivation of PA-Rac1 within individual cells resulting in a locally defined outgrowth of lamellipodial structures (Fig 3C). This approach allowed for a reliable quantification of lamellipodia dynamics using kymograph analysis (Fig 3D and 3E). Again, we did not observe significant alterations in lamellipodial kinetics in the absence of FMNL2, consistent with unchanged expression of critical components of lamellipodia formation like WAVE-1 or N-WASP [39, 40] (Fig 3F). In summary, these data support the notion of FMNL2 being dispensable for lamellipodia formation downstream of Rac1 in MCF10A cells. Accordingly, an impaired function of lamellipodia cannot explain the observed defects in the assembly of cell-cell contacts between FMNL2-/- cells.

### FMNL2 and Cdc42 both affect filopodia formation, but Cdc42 is dispensable for the formation of epithelial cell-cell contacts

**Fig 4:**
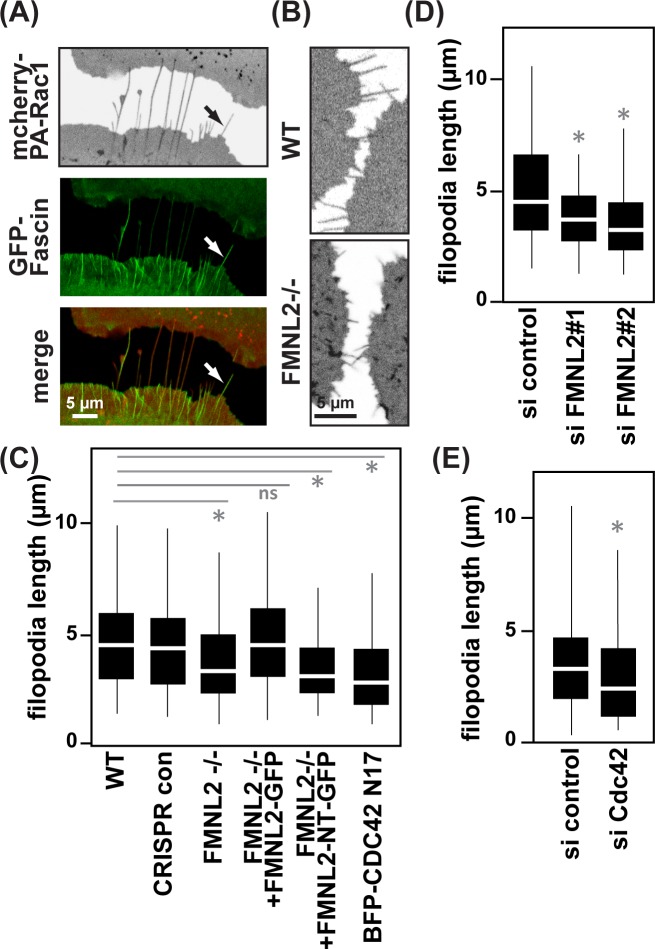
Cdc42 regulates filopodial growth and localization of FMNL2, but does not affect junction formation. **(A)** MCF10A cells co-expressing GFP-Fascin and mCherry-PA-Rac1. **(B)** Examples of filopodia being formed in WT or FMNL2-/- cells facing each other. **(C)** Quantification of filopodia length in indicated cell lines (*n* = 345 (WT), *n* = 448 (CRIPSR con), *n* = 364 (FMNL2-/-), *n* = 501 (FMNL2-/- + FMNL2-GFP), *n* = 408 (FMNL2-/- + FMNL2-NT-GFP), *n* = 185 (BFP-CDC42 N17), pooled from three independent experiments, *p≤0.001). **(D and E)** Quantification of filopodial length comparing FMNL2 or Cdc42 depleted cells to cells treated with control siRNA (*n* = 182 (si control), *n* = 61 (si FMNL2#1), *n* = 112 (si FMNL2#2), *n* = 274 (si control), *n* = 234 (si Cdc42), pooled from three independent experiments, *p≤0.01). Western blot demonstrating FMNL2 siRNA and Cdc42 siRNA efficiency is in S1G and S1J Fig.

Given its localization to filopodia tips and our impression of altered filopodia morphology in FMNL2 -/- cells (Fig 2F), we next tested for an impact of FMNL2 on filopodia dynamics. Prior to contact formation, we frequently observed prominent filopodial structures in cells facing each other, which were positive for the filopodia marker GFP-Fascin (Fig 4A). Since we found PA-Rac1 to faithfully label these structures and to avoid any potential alterations of filopodia dynamics arising from the overexpression of the F-actin bundling protein Fascin [41], we decided to further monitor and analyze filopodia based on the fluorescence signal produced by mCherry-PA-Rac1 (Fig 4B). Taking the length of filopodia prior to contact formation into account, we found a significantly reduced length in FMNL2 -/- cells (Fig 4C), comparable to expression of a dominant-negative variant of Cdc42 (Cdc42 N17), known to impair formation of filopodia [42, 43] (Fig 4C). Again, this phenotype could be fully reverted by the reexpression of full-length FMNL2 but not its inactive variant FMNL2 NT (Fig 4C). As a complementation to our previous publication and to exclude potential long-term effects due to genetic knockout, we confirmed our findings by siRNA-mediated depletion of FMNL2 or Cdc42 (Fig 4D and 4E).

### Rac1, but not Cdc42, recruits FMNL2 during native epithelial contact formation

**Fig 5:**
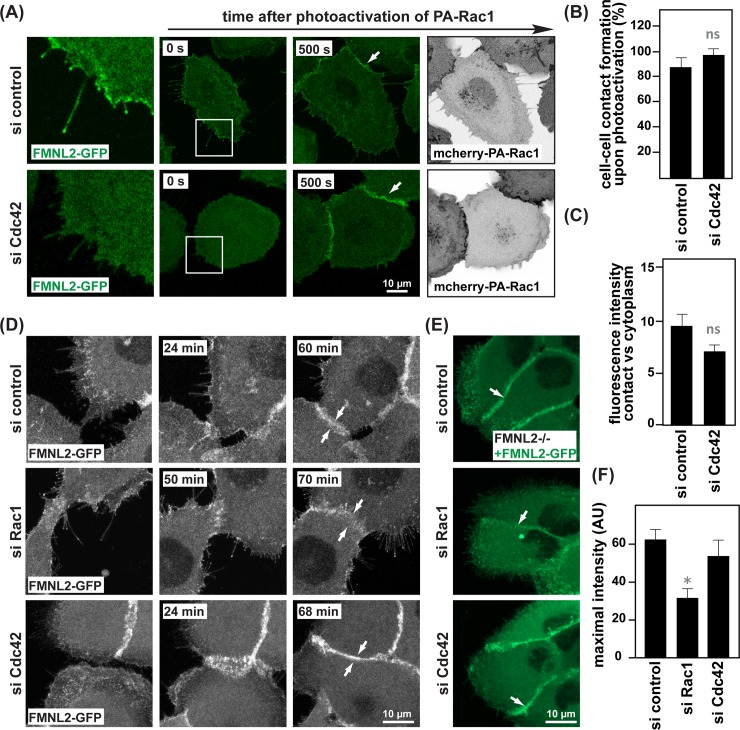
Rac1 is required for FMNL2 localization to cell-cell contacts. **(A)** Live-cell imaging of FMNL2-/- cells expressing FMNL2-GFP together with mCherry-PA-Rac1 transfected with either control siRNA or siRNA directed against Cdc42. Red arrows highlight FMNL2-GFP localization in filopodia **(B)** Quantification of cell-cell contact formation as in (A). (*n* = 53 (si control), *n* = 43 (si Cdc42), pooled from three independent experiments, ns non-significant, calculated by *t*-test). **(C)** Quantification of fluorescence intensity of FMNL2-GFP at cell-cell contacts versus cytoplasm (*n* = 13 (si control), *n* = 13 (si Cdc42), pooled from three independent experiments, ns non-significant, determined by *t-*test.) **(D)** Time lapse images of FMNL2-/- cells expressing FMNL2-GFP transfected with control, Rac1 or Cdc42 siRNA. **(E)** Stills of live FMNL2 -/- cells expressing FMNL2-GFP transfected with control, Rac1 or Cdc42 siRNA. Arrows highlight FMNL2-GFP at cell-cell contacts. **(F)** Quantification of fluorescence intensity at cell-cell contacts in cells treated with siRNA as indicated (*n* = 8 (si control), *n* = 12 (si Rac1), *n* = 6 (si Cdc42), pooled from three independent experiments,*p≤0.01, error bars SEM). siRNA efficiency in MCF10A cells is demonstrated in S1J Fig.

In light of previous reports demonstrating the ability of FMNL2 to interact with Cdc42 [22] and given the potential functional consequences in terms of FMNL2 localization and activation arising from this interaction, we wondered about a contribution of Cdc42 in FMNL2-mediated cell-cell contact formation. Consistent with a model of FMNL2 acting downstream of Cdc42 during filopodia extension, we observed a specific loss of FMNL2 localization to the tips of residual filopodia in the absence of Cdc42 (Fig 5A). This prompted us to further test for the importance of Cdc42 during contact formation. Surprisingly, neither depletion of Cdc42 nor expression of dominant-negative Cdc42 N17 altered the efficiency of MCF10A cells to undergo contact formation (Fig 5A and 5B, S1H Fig). Consistently, we did not observe a substantially impaired recruitment of FMNL2 to newly forming epithelial contacts under conditions of Cdc42 depletion (Fig 5A and 5C). Thus, our findings argue strongly against a Cdc42-mediated control of FMNL2 localization and function at the cell-cell interface.

To further study the cell-cell contact specific recruitment of FMNL2, we followed contact formation between MCF10A cells under entirely native conditions. Time-lapse imaging of MCF10A cells stably expressing FMNL2-GFP confirmed contact-specific recruitment of FMNL2 once cells encounter each other, without the need of experimental stimulation of Rac1 activity (Fig 5D, S3 Movie). Importantly, under these conditions targeting of FMNL2 was clearly attenuated in cells depleted for Rac1, whereas silencing of Cdc42 again had no noticable effect (Fig 5D). Of note, we also observed a similar and again specifically Rac1-dependent localization of FMNL2 to the sites of matured cellular contacts within established epithelial cell sheets (Fig 5E and 5F), strongly arguing for a functionality of FMNL2 at cellular junctions even beyond *de novo* assembly, independent of Cdc42, but specifically linked to Rac1 signaling. In line with this, our previous work revealed the ability of FMNL2 to alter junctional actin turnover with likely implications on the overall physical properties of epithelial cell-cell junctions [21].

## Discussion

Here, we report a cell-cell contact-specific activity and upstream regulation of the actin assembly factor FMNL2 downstream of Rac1. Our results indicate that functions of FMNL2 in the establishment and maintenance of cell-cell contacts cannot be attributed to defects in the formation of actin-based membrane protrusions, which were recently described for FMNL2 in migrating B16-F1 melanoma cells [23]. While confirming contributions of FMNL2 to the formation of filopodia [20, 22], our data exclude a significant impact of FMNL2 on lamellipodia dynamics in MCF10A human epithelial cells (Figs 3 and 4). Of note, we draw this conclusion based on a very reliable and minimalistic optogenetic system (induction of lamellipodia formation by photoactivation of PA-Rac1) which enabled us i) to locally stimulate membrane protrusion in a highly restricted and defined manner [21, 26] and ii) to study lamellipodia dynamics without any confounding signaling inputs arising e.g. from chemotactic sources during cell migration. However, our experiments cannot rule out potentially cell type-specific functions of FMNL2 within lamellipodial protrusion. In this regard, it should be noted that recent findings in epithelial cells suggest that they rather use protrusive membrane structures to explore their immediate surrounding than to migrate over longer distances [6]. Hence, epithelial cells might use alternative ways to extend Rac1- and Arp2/3-dependent protrusions, without major contributions of FMNL2.

Alternatively, closely related formins such as FMNL2 and FMNL3 could exhibit synergistic functions and cooperate within protrusive structures such as lamellipodia. Supporting this idea, FMNL2 and FMNL3 have been shown to heterooligomerize and the double knockout of FMNL2/FMNL3 in B16-F1 cells resulted in a stronger reduction of lamellipodial width and reduced F-actin intensity compared to single knockouts [23]. Therefore, it remains to be tested to what extent FMNL3 might compensate for the loss of FMNL2 during lamellipodia formation in MCF10A cells. Interestingly, FMNL3 has also been reported to function at cell-cell contacts where it regulates junctional actin dynamics and depletion of FMNL3 in wound healing assays resulted in reduced adhesion of cells at the migrating front [18, 44]. Future research is needed to quantitatively address the functional and physical properties of individual FMNL formins at cell-cell contacts in more detail.

In contrast to recent reports, we find FMNL2 functions to be entirely independent of Cdc42 [16, 22]. In fact, we not only confirm that Rac1 activation affects FMNL2 localization but also show that endogenous Rac1 is critical for the recruitment of FMNL2 to newly forming junctions as well as within already established epithelial sheets (Fig 5). While silencing of Cdc42 phenocopies the decreased filopodia length observed in FMNL2-/- cells, we do not find Cdc42 to affect localization or functions of FMNL2 at intercellular junctions (Figs 4 and 5). Therefore, our results indicate a regulatory promiscuity of FMNL2 upstream regulation through different Rho GTPases that occurs most likely context-dependent and in line with reports on other formins such as mDia1 [45, 46]. Accordingly GTPases might primarily function to specifically recruit FMNL2 to certain subcellular regions whereas its activity might rather be amplified by alternative signalling inputs and posttranslational modifications like for example the PKC-dependent phosphorylation within its C-terminus [36]. While the crystal structure of Cdc42 bound to the N-terminus of FMNL2 has been recently solved [22], the ability of either Cdc42 or Rac1 to control the activity state of FMNL2 through conformational release of autoinhibition still remains to be experimentally tested.

The specific and Rac1-dependent localization of FMNL2 to cell-cell contacts is intriguing. Our experiments suggest that the N-terminus of FMNL2 harbouring the Rho GTPase binding domain is critical for the localization to junctions and the C-terminal domain, containing the catalytically active FH2 domains, is required for functional rescue (Fig 2). Pull-down assays revealed that FMNL2 associates with E-Cadherin in a Rac1-dependent manner and the FMNL CT can be detected at the intracellular C-terminal part of E-Cadherin, a finding which is further supported by quantitative proteomic analysis [21, 47]. It remains to be explored how this association ultimately affects functionality of FMNL2 at cell-cell contacts. Of note, in vitro results indicate that this interaction can at least in part compete with the autoinhibitory binding between the FMNL2 N- and C-terminal regions [21]. On the other hand, binding of FMNL2 to E-Cadherin could as well contribute to the enrichment of FMNL2 at E-Cadherin-based cell-cell contacts. However, our live-cell experiments reveal a rather simultaneous enrichment of both E-Cadherin and FMNL2 at newly forming junctions.

Taken together, cell-cell contact-related functions as well as localization of FMNL2 cannot be attributed to previously described functions of FMNL2 in the formation of actin-based protrusions. Therefore, our work adds evidence to a complex and context-dependent regulation of formins involving multiple Rho GTPases and likely additional signaling inputs. In this regard, FMNL2 exemplifies versatile functionalities of a single formin protein, realized by coordinated upstream signaling and adapted to specific cellular needs.

## Supporting information

S1 Fig **(A)** Exon 2 of FMNL2 including the guide sequence and protospacer adjacent motif (PAM). **(B)** Example of Sanger sequencing of wildtype and FMNL2-/- FMNL2 exon two. Please note, the shown sequence refers to at least one allele of the FMNL2 gene. Red asterisk indicates the Cas9-induced deletion in the CRISPR cell line. Red asterisk indicates the Cas9-induced deletion in the CRISPR cell line. **(C)** Putative amino acid sequence of WT and FMNL2 -/-. The microdeletion in exon 2 likely leads to a frame shift resulting in a premature stop codon (red asterisk). **(D)** Example T7 Assay revealing Cas9-induced mutation in FMNL2 exon 2. **(E)** Western blot confirming efficient siRNA knockdown of E-Cadherin. Tubulin served as a loading control. **(F)** HEK cells expressing FMNL2 variants were subjected to SRF luciferase assay. Titration of FMNL2-NT-GFP to the active FMNL2 C-terminus led to an expected reduction of luciferase activity. (N = 3, error bars indicate SD). **(G)** Western blot showing knockdown efficiency after FMNL2 siRNA treatment. **(H)** Localization of BFP-Cdc42 N17 (blue channel) and mCherry-PARac1 (red channel) in fixed MCF10A cells. Western blot showing the inducible expression of BFP-Cdc42 N17. **(I)** Quantification of cell-cell contact formation after induction of BFP-Cdc42 N17 N17 (*n* = 16 (con), *n* = 44 (BFP-Cdc42 N17), pooled from two different experiments, *p* values were calculated by *t*-test)**. (J)** Western blot showing the knockdown efficiency of Cdc42 and Rac1 siRNA.
(TIF)

S1 Movie MCF10A FMNL2-/- cells stably expressing FMNL2-GFP and mCherry-PA-Rac1 were monitored over time using time-lapse confocal microscopy. Note Rac1-acitvity is continuously uncaged by the 488-nm laser.
(AVI)

S2 Movie MCF10A FMNL2-/- cells stably expressing FMNL2-GFP and mCherry-PA-Rac1 were monitored over time. Note that, PA-Rac1 is activated through the 488-nm laser, also exciting the FMNL2 NT-GFP signal.
(AVI)

S3 Movie Cell-cell contact formation in MCF10A FMNL2-/- cells expressing FMNL2-GFP was monitored over time. Cells were transfected with indicated siRNAs.
(AVI)
