# Supplementary figures and images for: A Rac1-FMNL2 signaling module affects cell-cell contact formation independent of Cdc42 and membrane protrusions
Source: PLoS One. 2018 Mar 26;13(3):e0194716. doi: 10.1371/journal.pone.0194716 (PMC5868805; doi:10.1371/journal.pone.0194716)

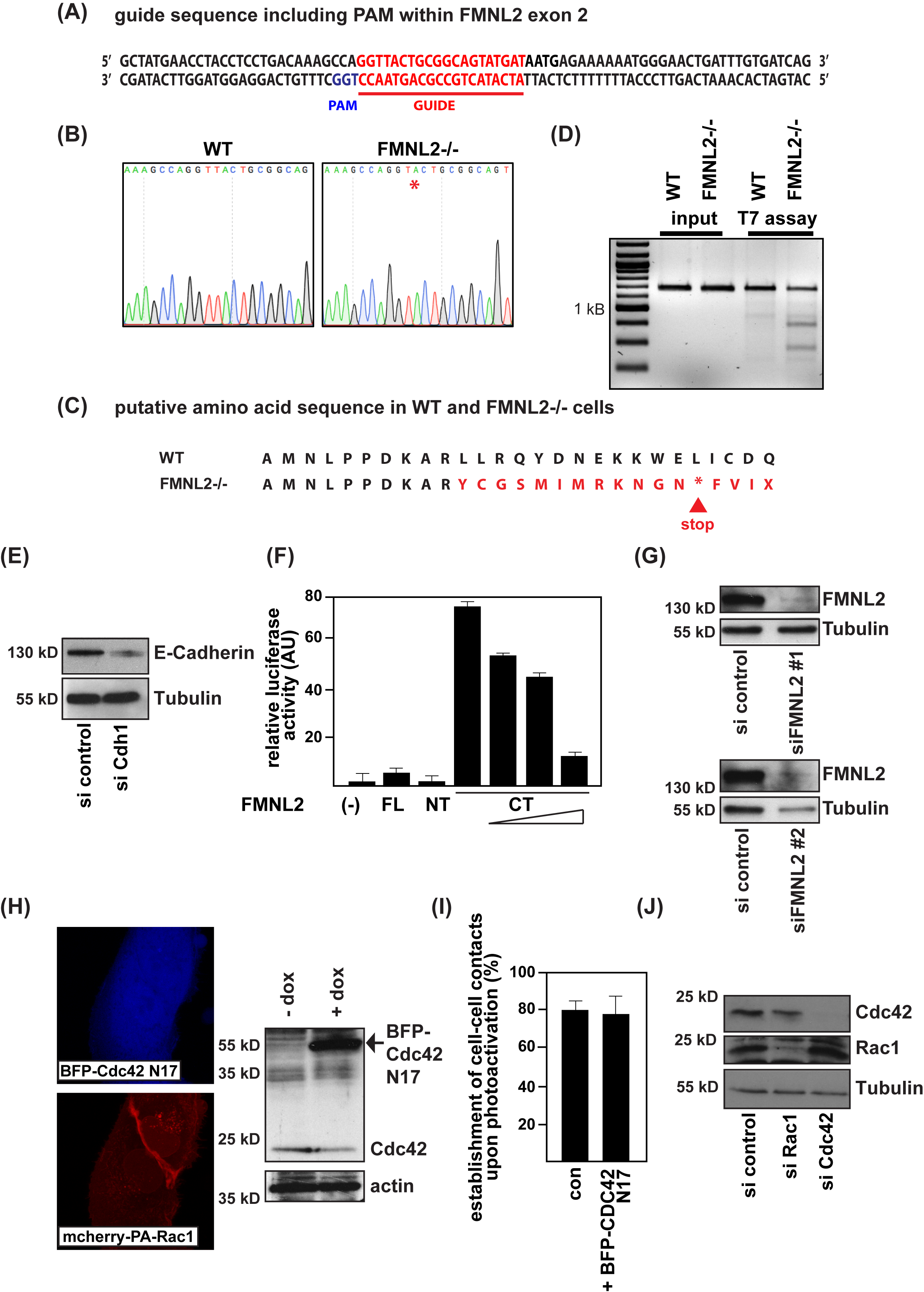

Supplement: S1 Fig — (A) Exon 2 of FMNL2 including the guide sequence and protospacer adjacent motif (PAM). (B) Example of Sanger sequencing of wildtype and FMNL2-/- FMNL2 exon two. Please note, the shown sequence refers to at least one allele of the FMNL2 gene. Red asterisk indicates the Cas9-induced deletion in the CRISPR cell line. Red asterisk indicates the Cas9-induced deletion in the CRISPR cell line. (C) Putative amino acid sequence of WT and FMNL2 -/-. The microdeletion in exon 2 likely leads to a frame shift resulting in a premature stop codon (red asterisk). (D) Example T7 Assay revealing Cas9-induced mutation in FMNL2 exon 2. (E) Western blot confirming efficient siRNA knockdown of E-Cadherin. Tubulin served as a loading control. (F) HEK cells expressing FMNL2 variants were subjected to SRF luciferase assay. Titration of FMNL2-NT-GFP to the active FMNL2 C-terminus led to an expected reduction of luciferase activity. (N = 3, error bars indicate SD). (G) Western blot showing knockdown efficiency after FMNL2 siRNA treatment. (H) Localization of BFP-Cdc42 N17 (blue channel) and mCherry-PARac1 (red channel) in fixed MCF10A cells. Western blot showing the inducible expression of BFP-Cdc42 N17. (I) Quantification of cell-cell contact formation after induction of BFP-Cdc42 N17 N17 (n = 16 (con), n = 44 (BFP-Cdc42 N17), pooled from two different experiments, p values were calculated by t-test). (J) Western blot showing the knockdown efficiency of Cdc42 and Rac1 siRNA. (TIF) [file pone.0194716.s001.tif]
